# Neurophysiological insights into sit-to-stand post stroke

**DOI:** 10.3389/fnins.2025.1646498

**Published:** 2025-09-02

**Authors:** Caitlin McDonald, John Jairo Villarejo Mayor, Olive Lennon

**Affiliations:** School of Public Health, Physiotherapy and Sports Science, University College Dublin, Dublin, Ireland

**Keywords:** stroke, sit-to-stand, EMG, EEG, corticomuscular coherence

## Abstract

**Introduction:**

Stroke often results in the loss of ability to stand-up independently or to perform the transfer with compensatory movement patterns. While neurological disorders are associated with sit-to-stand disability, the neurophysiological mechanisms underlying the movement and the impact of injury at brain level remain poorly understood.

**Methods:**

Stroke participants (*n* = 10, 4 males) performed five sets of three sit-to-stand transitions from an armless, backless seat adjusted to their knee joint height with three-dimensional kinematic data capture. Electromyography (EMG) was recorded from the bilateral vastus lateralis, biceps femoris, tibialis anterior, and gastrocnemius muscles. Surface electroencephalography (EEG) activity was recorded using eight focused bipolar channels over the sensorimotor cortex. Data were analyzed and compared with a reference dataset from healthy adults (*n* = 10).

**Results:**

Kinematic data confirms post-stroke participants take significantly longer to complete a sit-to-stand transfer compared to healthy controls but maintain the same kinematic movement phases and temporal muscle activation patterns. EMG data indicates stroke survivors stand up using largely the same temporal muscle activation patterns, however they exhibit delayed peak activity of the vastus lateralis and biceps femoris compared to healthy controls. EEG data reveal stroke survivors demonstrate variable event-related spectral perturbation patterns and reduced event-related synchronization/de-synchronization in the alpha and beta frequency bands and increased asymmetry between brain hemispheres when compared to healthy controls.

**Conclusion:**

EMG data supports the wider literature that confirms stroke survivors stand up using the same temporal muscle activation patterns compared to healthy controls, however peak activity of the vastus lateralis and biceps femoris are delayed. EEG data add new knowledge to our understanding of the central control of sit-to-stand transfers in a stroke population, highlighting differences in cortical activity from healthy controls, notably in ERSP patterns during sit to stand phases and in brain hemisphere asymmetry. Findings have relevance as a potential biomarker for stroke functional recovery and indicate that BCI-based applications of sit to stand may need to be trained individually in stroke survivors as they demonstrate variable cortical activation patterns compared to healthy controls.

## 1 Introduction

Stroke, as defined by the World Health Organization, is an accident to the brain with “rapidly developing clinical signs of focal or global disturbance to cerebral function, with symptoms lasting 24 h or longer, or leading to death, with no apparent cause other than of vascular origin” (Aho et al., 1980). Stroke is the second leading cause of death, and the third leading cause of death and disability combined worldwide (Feigin et al., 2022). Persistent physical disability is reported in 50–65% of individuals who survive stroke (Mudge and Stott, 2007). Hemiplegia, from damage in one hemisphere of the brain, is one of the most common physical impairments after stroke, resulting in contralateral loss of muscle function, and altered tone, sensation and reflexes (Olney and Richards, 1996; Belda-Lois et al., 2011; Flansbjer et al., 2005). Many stroke survivors do not reach a level of mobility that enables them to perform all previous activities of daily living, with approximately 16% unable to stand-up independently post-stroke (Harrison et al., 2013; Logan et al., 2022; Geyh et al., 2004). Stroke survivors who can stand up independently commonly demonstrate an altered sit-to-stand movement pattern (McDonald et al., 2024) with higher energy expenditure and a higher risk of falls (Cheng et al., 1998; Harington et al., 2024). Reduced peak vertical ground reaction force, increased medio-lateral center of pressure displacement, and altered weight distribution between the lower limbs are commonly observed during the sit-to-stand transfer following stroke (Cheng et al., 1998; Boukadida et al., 2015; Pollock et al., 2014). The overall time required to complete the movement is also prolonged (Hsu et al., 2019). Rehabilitation interventions often focus on reducing sit-to-stand duration and improving lateral symmetry, by promoting more balanced weight distribution between the legs during the task (Pollock et al., 2014).

Despite these compensations, kinematic investigations have shown no statistically significant differences between the sit-to-stand kinematics of individuals post stroke and those of healthy controls (Hsu et al., 2019). The four distinct phases of sit-to-stand (flexion momentum, momentum transfer, extension and stabilization) remain consistent across older and younger populations, as well as neurological subgroups including stroke (Schenkman et al., 1990; Hanawa et al., 2017). Similarly, the three muscle synergies acknowledged in the literature as required to reconstruct lower-limb muscle activation during the sit-to-stand transfer (McDonald et al., 2024; Hanawa et al., 2017) (tibialis anterior and quadriceps/quadriceps, hamstrings, and glutes/soleus and gastrocnemius) remain evident even in the presence of central pathologies like stroke (McDonald et al., 2024; Hsu et al., 2019; Wang et al., 2022). However, overall sit-to-stand movement time is prolonged following stroke, accompanied by increased muscle activity sustained over a longer duration, a greater reliance on the unaffected limb and delayed onset timings of muscles in the affected limb (Hsu et al., 2019; Prudente et al., 2013). No brain activity data in individuals post stroke during 3D kinematics of sit-to-stand were identified.

Brain activation during sit-to-stand however is poorly understood especially in neurological populations such as stroke survivors despite their association with the inability to sit-to-stand independently (Boukadida et al., 2015). A recent systematic review in the area identified no studies collecting and reporting co-registered 3D kinematic and EEG data during active sit-to-stand in healthy or pathological populations (McDonald et al., 2024). Under-exploration of EEG activity during sit-to-stand activity is due, in part, to practical limitations that include significantly higher EEG artifact during complex movements like sit-to-stand and limitations in older wire based systems (Kline et al., 2021). As a result, EEG research during sit-to-stand has focused mostly on imagined movement; classification of different lower limb movements that include standing up and identification of the intention to stand up prior to execution (McDonald et al., 2024; Jochumsen and Niazi, 2020). An early study reporting brain activity during sit-to-stand transitions identified beta wave (14–17 Hz) activity in healthy adults during the transfer, though pre-movement and movement phase details were not described (Kanokwan et al., 2019). More recently, an investigation into the subphases of sit-to-stand observed a loss in beta amplitude [Event-Related Desynchronization (ERD)] in preparation to stand up and in the flexion and early extension phases. This was followed by an amplitude rebound above baseline [Event-Related synchronization (ERS)] or ‘overshoot’ when upright standing was achieved (McDonald et al., 2025). It remains to be seen whether these frequency modulations remain consistent in stroke survivors. Evidence from EEG recordings and functional magnetic resonance imaging (fMRI) during other functional movements do reveal differences in cortical activity between healthy controls and stroke survivors, with stroke survivors showing asymmetry between brain hemispheres (Riecker et al., 2010) and weaker ERD (Peter et al., 2022). It has been suggested that this power reduction is associated with impaired cortical activation and motor deficits (Tang et al., 2020).

Cortico-muscular coherence (CMC) reflects the functional coupling between cortical oscillatory activity and muscle output, most commonly observed in the beta frequency range, and serves as a marker of sensorimotor integration (Liu et al., 2019). Stroke patients have been shown to exhibit significantly lower CMC compared to healthy controls in both the beta (20–30 Hz) and lower gamma (30–40 Hz) bands during movement (Fang et al., 2009). Interestingly, even among healthy individuals, the presence of CMC during functional tasks such as sit-to-stand has been inconsistently observed (McDonald et al., 2025), highlighting the need to further investigate its role in neurologically impaired populations.

The influence of altered cortical activity on compensatory movement patterns during sit-to-stand in individuals post-stroke remains poorly understood, due to a lack of research examining brain activity during the execution of sit-to-stand in this population. This study aims to better understand the neurophysiological basis of sit-to-stand after stroke by analyzing synchronized EEG, EMG, and kinematic data across different phases of the movement and evaluating the variances between recordings in healthy individuals and post-stroke participants.

## 2 Methods

### 2.1 Participants

Participants were volunteers by self-selection recruited through poster campaigns in the university, local retirement groups and Irish Heart Foundation community stroke support centers. Self-selection indicates that individuals chose to participate on their own initiative, without being directly invited or selected by the researchers. Participants were included if they were over 18 years of age, had no known neurological condition (other than stroke) and could stand independently (permitting the use of mobility aids) from a regular height surface. All stroke types and locations were considered eligible for inclusion, as were participants with any number of previous stroke occurrences. Participants were excluded if they presented with a physical impairment that limited data collection (modified Rankin Scale > 4) (Pożarowszczyk et al., 2023) or a cognitive impairment that limited their ability to provide informed consent (COGTEL score < 15, subject to further investigation) (Ihle et al., 2018). A COGTEL score below 15 was considered indicative of severe cognitive impairment and prompted further assessment to determine capacity to provide informed consent. For participants scoring below this threshold, additional capacity assessments were conducted in line with ethical guidelines. Individuals who demonstrated sufficient understanding, appreciation, reasoning, and ability to express a consistent choice, were deemed capable of providing informed consent. Informed and written consent was obtained from each subject prior to participation. The Human Research Ethics Committee at University College Dublin, the National University of Ireland approved the experimental protocols (LS-22-19-Lennon), conducted in accordance with the Declaration of Helsinki.

The comparator group consisted of 11 age and sex-matched healthy adults. Healthy participants had no known neurological conditions or musculoskeletal impairments that would impact data collection. These participants were selected from a larger dataset collected as part of a previous study in healthy participants (McDonald et al., 2025), ensuring sex matching and the closest possible age match available. An identical data collection protocol was employed in both studies.

### 2.2 Data collection protocol

On arrival to the motion analysis laboratory each participant’s height, weight Functional Ambulation Category (FAC) (Mehrholz et al., 2007) and modified Rankin Scale (mRS) for neurological disability (Pożarowszczyk et al., 2023) were recorded by a research physiotherapist. Participants were positioned on an armless, backless seat, adjusted in height to the knee joint height of each participant with the knee at 90 degrees flexion. Knee joint angle estimation was performed via visual observation by a physiotherapist. Initially a two-minute resting EEG trial was recorded. This was followed by a target of five sets of three sit-to-stand and stand-to-sit transitions at a self-selected pace, pausing for approximately 5 s between each transition. In cases where participants were unable to complete all trials due to fatigue, data collection concluded at that point. Participants were instructed to stand up and sit down in a natural way with the minimal use of upper limb assistance. Participants were permitted to use any mobility aids routinely employed in daily life to perform sit-to-stand transfers. To minimize movement artifact in EEG recordings, participants were asked to avoid excessive blinking, teeth grinding and jaw clenching. To ensure familiarity with the task and consistency across participants they performed a practice trial of 3 sit-to-stand repetitions with verbal correction or demonstrations provided where required.

### 2.3 Data collection equipment

Electroencephalography was recorded at a sampling frequency of 250 Hz using a wireless system of eight bipolar channels (FlexEEG, NeuroCONCISE, UK) over the sensorimotor cortex [C6, C4, C2, Cz, FCz, C1, C3, and C5 locations defined by the international 10/20 system of electrode placement (Klem et al., 1999)]. Electrode Afz was used as a reference. Conductive gel was used to maintain low electrode impedance. Impedance measurement was not conducted, as it was not supported by the recording system used. Data were recorded and saved using Matlab software (Mathworks, USA).

Electrical muscle activity was measured using eight wireless EMG electrodes with a 2000-Hz sampling rate (Delsys, Trigno, USA). Electrodes were placed bilaterally on vastus lateralis (VL), biceps femoris (BF), tibialis anterior (TA), and gastrocnemius (GAS) muscle groups according to SENIEM guidelines (Stegeman and Hermens, 2007).

A 3D motion capture camera system was used to record the kinematic joint angles (Codamotion 3D system, UK). Four cameras captured the movement by active infrared retroreflective wearable marker clusters, attached to both sides of the body, on the pelvis, upper leg, lower leg, calcaneus, and fifth metatarsal bases, and one marker cluster was placed in the center of the chest. In addition, reflective markers were attached bilaterally to anatomical landmarks (anterior superior iliac spine, lateral femoral epicondyle, and medial femoral epicondyle; lateral and medial malleoli) and the right acromioclavicular joint, to create a three-dimensional whole-body segment model. Kinematic data were recorded at a 100-Hz sampling rate. Hip, knee and ankle joint angles were estimated from spatial position data by the Codamotion ODIN software platform (ODIN, Codamotion, UK).

All EEG, EMG and ODIN data were synchronized. EMG data was integrated digitally with the kinematic data via Coda-Hub. synchronization of the FlexEEG system with Coda-Hub was achieved using a Delsys Trigger Module (Delsys, USA) and a LabJack U12 USB DAQ module (LabJack, USA). When kinematic recording began, a pulse from the trigger module was sent to Coda-Hub to start EMG recording, and a second pulse was sent from Coda-Hub to Matlab (Mathworks, USA) through the LabJack module via USB.

### 2.4 Data processing

The sit-to-stand movement was divided into two broad dynamic phases based on kinematic joint angle data (collapsing the four known phases of sit-to-stand to two). Phase one, the flexion phase, began with the initial detection of hip flexion movement, which was manually identified by visual inspection of the hip joint angle time series as the first increase in flexion, and ended at the point of maximum hip flexion (combining upper body forward momentum and pelvic de-weighting phases). Phase two, the extension phase, began at maximum hip flexion and ended at maximum hip extension (combining the extension and stabilization phases). Pre and post movement activity were recorded in quiet sitting, 0.5 s prior to the beginning of the flexion phase, and in upright standing 0.5 s after termination of the extension phase.

EMG data were bandpass filtered between 20 to 250 Hz (zero-phase 2nd -order Chebyshev). A 50 Hz notch filter was used to remove power-line interference. The offset for each channel was removed by subtracting the trial signal average (Yin et al., 2020; Konrad, 2005).

Raw EEG were bandpass filtered, 0.1 to 60 Hz with a zero-phase 2nd -order Chebyshev. A notch filter centered at 50 Hz with a 2 Hz bandwidth was used to remove power line interference. EEG data were then submitted to an automated artifact rejection procedure, Artificial Space Removal (ASR) using the EEGLAB toolbox (clean_artifacts) in Matlab (Mathworks, USA) to remove artifacts related to low frequency drifts, noisy channels and short-time bursts. The input arguments used were: ‘BurstCriterion’ configured as ‘20’; ‘WindowCriterion’ and ‘ChannelCriterion’, configured as ‘off’ to avoid deleting data windows and channels; and ‘BurstCriterionRefMaxBadChns’ to pass the calibration data. For each participant, ASR was calibrated from the EEG data collected during the 2-min resting state condition and applied to all the movement trials performed, in line with previous studies (Tortora et al., 2020; Tortora et al., 2023; Artoni et al., 2017). The resting period was used instead of the more common task-specific period as the short trial duration and movement artifact limited calibration. Segments with absolute voltages above 100 μV were removed. Pre-processed EMG and EEG data underwent manual visual inspection. EMG and EEG channels were inspected in the time frequency domain and those with high level of artifact that were deemed not to contain meaningful information were excluded from analysis (Supplementary Table S1). Epochs that lacked characteristic EMG/EEG features such as well-defined activation/oscillatory patterns and consistent baseline activity were excluded. Signals with abrupt, irregular deflections, non-physiological flat-lining, high-frequency noise inconsistent with neural activity were flagged as likely artifacts and discarded. This manual inspection process ensured the retention of only physiologically plausible bio-signal data for further analysis.

### 2.5 Data analysis

Data sets were individually screened for quality of EMG and EEG signals. In the case where one of the two inputs was not viable or deemed not to be of sufficient quality, that entire sit-to-stand trial was discarded. Participants were excluded from analysis if they did not reach a minimum of 6 viable sit-to-stand transitions.

ERSP was calculated over the movement phases, to measure the modulation of the EEG activity in the frequency domain across the two sit-to-stand phases delineated. Time-frequency decomposition was performed on each channel and trial individually, followed by averaging across trials at each frequency. For normalization, the mean power across the entire task duration was computed for each frequency and used as a baseline. This method is well-suited for dynamic motor tasks, as it emphasizes time-varying spectral modulations relative to the overall task-related activity (Tortora et al., 2023; Gwin et al., 2010). This analysis was performed using the EEGLAB toolbox (newtimef), providing the epoch times at which the phases occur in each trial using the argument ‘timewarp’, and setting the ‘trialbase’ argument to ‘full’ to perform single trial normalization. These settings were used to align the timepoints of each phase for the different trials. Power spectral density (PSD) was computed from the ERSP for each phase of sit-to-stand in delta (0.5-4 Hz), theta (4–8 Hz), alpha (8–14 Hz), beta (14–30 Hz) and gamma (30–40 Hz) power bands, normalized to their average power. Topographic maps were generated using the EEGLAB toolbox (topoplot) applying a mask for the surrounding canals (T7, T8, Fz, Pz, P4, P3, F3, and F4).

The EMG envelope was obtained by estimating the root-mean-square (RMS) amplitude using 250 ms sliding windows, overlapping by 50 ms. The EMG signal corresponding to each phase was timewarped using linear interpolation (0.1% resolution), to align the timepoints for averaging the different trials. RMS envelopes were normalized on an individual basis to the maximum value in amplitude found within each set (of three or five trial repetitions) to reduce inter-participant variability.

To analyze corticomuscular coherence (CMC), we examined the frequency-domain coupling between the EEG electrode Cz, which is located over primary motor cortical area, and each of the eight recorded leg muscles. We chose this centrally located sensor because it is widely employed to assess CMC during gait (Jensen et al., 2019; Gennaro and de Bruin, 2020; da Silva Costa et al., 2024) and it was found to show maximal beta-band CMC amplitudes (Spedden et al., 2019). On account of individual trial durations of the sit-to-stand phases being too short to allow for reliable spectral analysis, EMG and EEG data from all trials were concatenated separately for each movement phase by stacking all repetitions of the corresponding phase across trials. This resulted in a continuous dataset per phase that aggregated neural and muscular activity from multiple instances of the same movement segment. This approach was used, as an alternative to averaging, to improve spectral resolution (Johnson and Shinohara, 2012; Gangwani et al., 2023). A low pass filter, 100 Hz with a zero-phase 2nd -order Chebyshev, was then applied on both EMG and EEG data. EMG data was full-wave rectified prior to coherence analysis. The Matlab coherence function, with a Hamming window of 512 and overlapping of 192 samples, was used for CMC calculation. The significance of the coherence was assessed through surrogate data analysis by generating 75 surrogate coherence spectra [consistent with the companion paper on healthy controls (McDonald et al., 2025)] for each participant. These surrogates were created from random signals of the same length as the original signals to preserve relevant properties (Faes et al., 2004). A cumulative density distribution with the surrogate coherence peaks were built to compare against the real coherence peaks for statistical significance (*p* < 0.05) (Piitulainen et al., 2015).

### 2.6 Statistical analysis

Data are reported separately for stroke participants and healthy controls. For stroke participants, we refer to the stroke side as the lesioned brain hemisphere, i.e., the left stroke side refers to right limb impairment, and the right stroke side refers to left limb impairment.

Stroke participants’ hemiplegic side, or affected lower limb, were compared with the same lower limb of their matched healthy control participant during comparative analyses performed, detailed below.

Comparative analyses were performed between the ERSP average of healthy controls and stroke survivors for each of the individual movement phase of sit-to-stand in the alpha and beta frequency bands and the Cz, C1 and C3 EEG channels. These electrodes were selected because a previous sit-to-stand investigation in healthy controls indicated these locations were likely to show the largest changes in activity (McDonald et al., 2025). The Shapiro–wilk test was conducted to assess normality and subsequently select appropriate statistical analysis methods. The independent T-test was performed for data that was found to be normally distributed, and the Wilcoxon rank sum test was performed in cases where data was found to be not normally distributed. The significance level was set at *p* < 0.05.

Statistical comparison of inter-hemisphere ERSP (C1 and C3 electrode compared to C2 and C4 electrode) of both groups was completed using the paired T-test for data that was found to be normally distributed, and the Wilcoxon signed-rank test in cases where data was found to be not normally distributed. In the case of right hemisphere strokes the C2 and C4 electrodes were compared to the C1 and C3 electrodes, while for left hemisphere strokes the C1 and C3 electrodes were compared to the C2 and C4 electrodes. Further subgroup analysis was performed using the same methods, dividing stroke survivors by their level of impairment as indicated by the mRS. Correction for multiple comparisons was not applied, as the statistical analysis focused on a small number of planned comparisons, remained exploratory in nature, and was intended to inform future hypothesis-driven research.

## 3 Results

A total of 11 stroke survivors completed the data collection protocol. One participant’s data (participant 6) was excluded from analysis as a result of dataset viability issues, as detailed in Table 1. Thus, data from 10 post-stroke participants (3 males) with a mean age of 44 ± 9 years, height 173 ± 9 cm and weight 74.3 ± 10.3 kg are presented. Seven participants had left hemisphere strokes, two right hemisphere strokes and two had bilateral strokes. In seven cases the cause of stroke was ischaemic and three haemorrhagic. The average time since stroke was 4.8 years (± 2.8 years). Six participants had an mRS score of 1, indicating no significant physical disability, three had a score of 2 indicating some physical disability and one participant had a score of 3 reflecting they had a moderate physical disability (Pożarowszczyk et al., 2023). 8 participants had an FAC of 5 and two had an FAC of 4, indicating independent ambulation on any surface and stairs, and independent walking on level surfaces only, respectively (Mehrholz et al., 2007). A total of 118 sit-to-stand repetitions were included across the 10 post-stroke participants included in the analysis.

**Table 1:** Demographic information for stroke and matched control participants.

Participant	Group	Sex	Age (years)	Height (cm)	Weight (kg)	BMI (kg/m^2^)	Stroke type	Stroke laterality
1	Stroke	F	50	163.5	130	48.7	Ischemic	Right
1	Control	F	55	174.5	78	25.6	–	–
2	Stroke	F	36	180	108	33.3	Ischemic	Left
2	Control	F	35	172	100	33.8	–	–
3	Stroke	F	38	180	82	25.3	Ischemic	Left
3	Control	F	38	166.5	66	23.8	–	–
4	Stroke	F	42	172	98	33.1	Haemorrhagic	Right
4	Control	F	70	162	78	29.7	–	–
5	Stroke	F	33	165	102	37.5	Ischemic	Left
5	Control	F	28	165	64	23.5	–	–
6	Stroke	M	34	179	99	30.9	Ischemic	Left
6	Control	M	30	177	63	20.1	–	–
7	Stroke	M	55	180	108	33.3	Haemorrhagic	Left
7	Control	M	24	186	85	24.6	–	–
8	Stroke	F	40	162.5	72	27.3	Ischemic	Left
8	Control	F	70	160.5	88	34.1	–	–
9	Stroke	F	60	170	65	22.5	Ischemic	Left
9	Control	F	69	171	63.5	21.7	–	–
10	Stroke	M	55	190	119	32.9	Haemorrhagic	Bilateral
10	Control	M	42	172	64	21.6	–	–
Total	Stroke	4 M/7F	43.5 ± 9.2	173.3 ± 8.8	96.6 ± 19	31.9 + − 7.2	7 Ischemic/4 Haemorrhagic	2 Right/ 8 Left/ 1 Bilateral
Total	Control	4 M/7F	45.77 ± 18.4	170.9 ± 6.9	170.9 ± 6.9	25.7 + − 4.9	**-**	**-**
*p*-value			0.5	0.21	0.002	0.005		

The healthy control comparator group consisted of 11 sex (3 males) and age-matched (46 ± 18 years) adults whose data were collected as part of a previous study (McDonald et al., 2025) using an identical data collection protocol. Further detail on included trials and any electrodes removed from subsequent analysis are detailed in Supplementary Table S1. A detailed description of participants with a diagnosis of stroke including time since stroke, mobility aids used and mRS, FAC, COGTEL scores, can be found in Supplementary Table S2.

### 3.1 Kinematics

**Figure 1:**
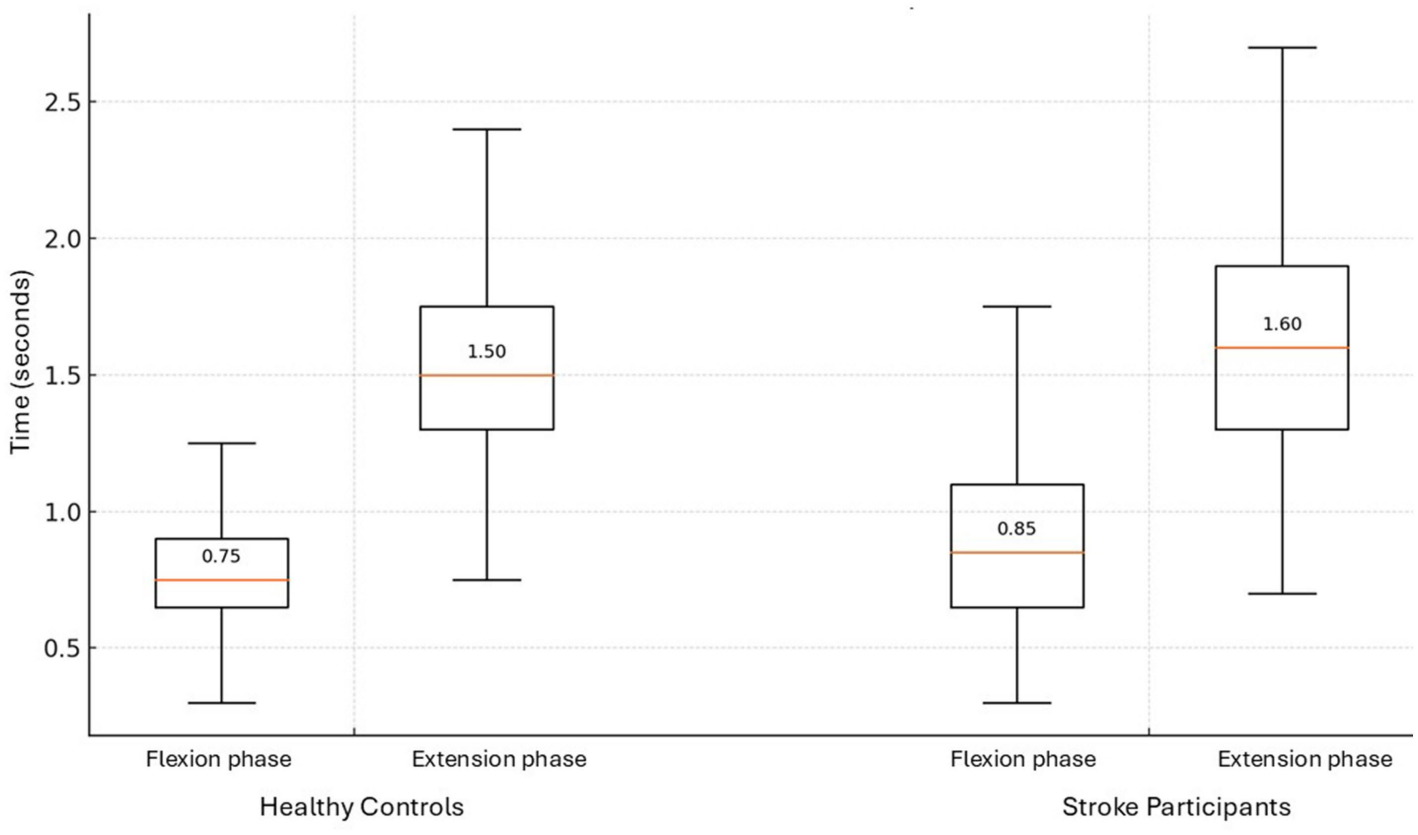
Phases of sit-to-stand transfer in stroke survivors and healthy controls.

Post-stroke participants took significantly longer to complete a sit-to-stand transfer (2.45 s) compared to healthy controls (2.25 s) (*p* = 0.038). There were no significant differences however in the temporal parameters of the movement phases between stroke and healthy individuals, when considered as two dynamic movement phases, the sit-to-stand transfer comprised 33% as the flexion phase and 67% as the extension phase in both groups. In the early flexion phase the hips and ankles were observed to begin to flex to initiate the movement. Hip flexion then continued and the hip was unloaded from the chair with some continuing knee and ankle flexion evident. In the extension phase the hips and knees were observed to begin to extend, coinciding with ankle plantar flexion. Toward the end of the extension phase as the body sought stability in upright standing, small joint adjustments were seen largely at the ankle joint. This is illustrated in Figure 1.

### 3.2 Muscle activity

**Figure 2:**
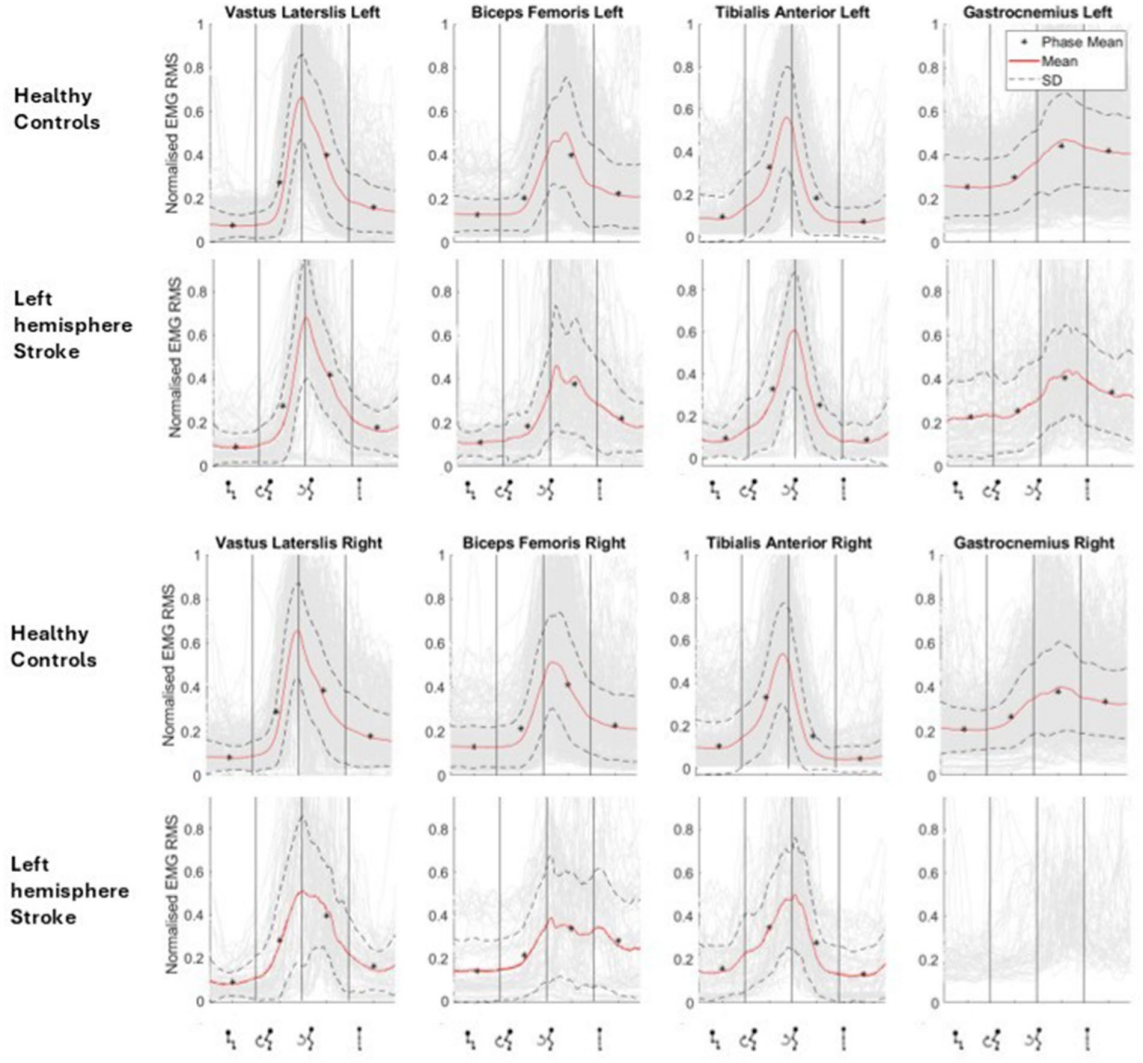
Muscle activation (as normalized RMS EMG) across phases of sit-to-stand (sitting prior to movement, flexion phase, extension phase and upright standing) averaged across all left hemisphere stroke participants compared to their matched healthy controls.

Figure 2 graphically depicts the average lower-limb muscle forces (RMS) of the four lower-limb muscles across sit-to-stand movement phases. Tibilalis anterior was activated first in the flexion phase, closely followed by vastus lateralis and biceps femoris. All three muscles reached peak EMG activity at seat off, the moment when the hip is unloaded from the chair and the hip joint angle trajectory changes from flexion to extension, i.e., the transition point between the flexion and extension phases. Across these three muscle groups, activity then reduced until upright standing was achieved, when activity plateaued marginally above (~0.5 RMS) baseline sitting activity. The gastrocnemius was most active in the extension phase and remained active in upright standing. The muscle that reached the highest level of normalized RMS activity was the vastus lateralis. This pattern was largely consistent with that observed in healthy controls however, the stroke group exhibited peak EMG activity at later points during the transfer. Delays were observed in the bilateral vastus lateralis (left: 0.32 s and right: 0.16 s) and in the right biceps femoris (0.425 s), relative to the healthy controls.

Figure 2 shows muscle activation (as normalized RMS EMG) across phases of sit-to-stand averaged across all left hemisphere stroke participants compared to their matched healthy controls.

### 3.3 Cortical activity

**Figure 3:**
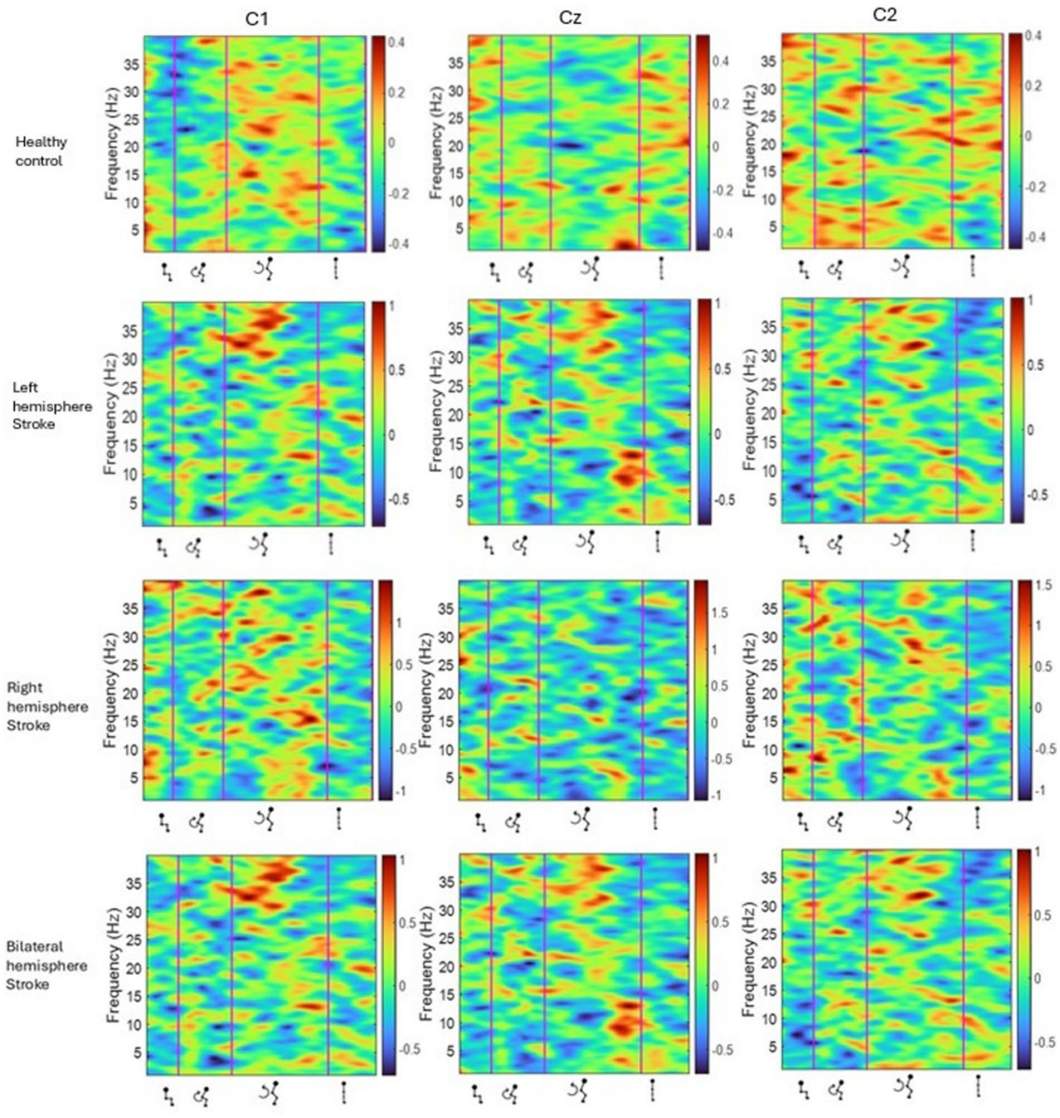
ERSP of one healthy control participant (P1), one participant with left hemisphere stroke (P10), one participant with right hemisphere stroke (P4) and one participant with a bilateral stroke (P11). Y-axis frequency between 0 and 40 Hz, and in x-axis the time in milliseconds and kinematic phases of sit-to-stand. Red and yellow spots represent synchronization, and darker blue spots represent desynchronization.

**Figure 4:**
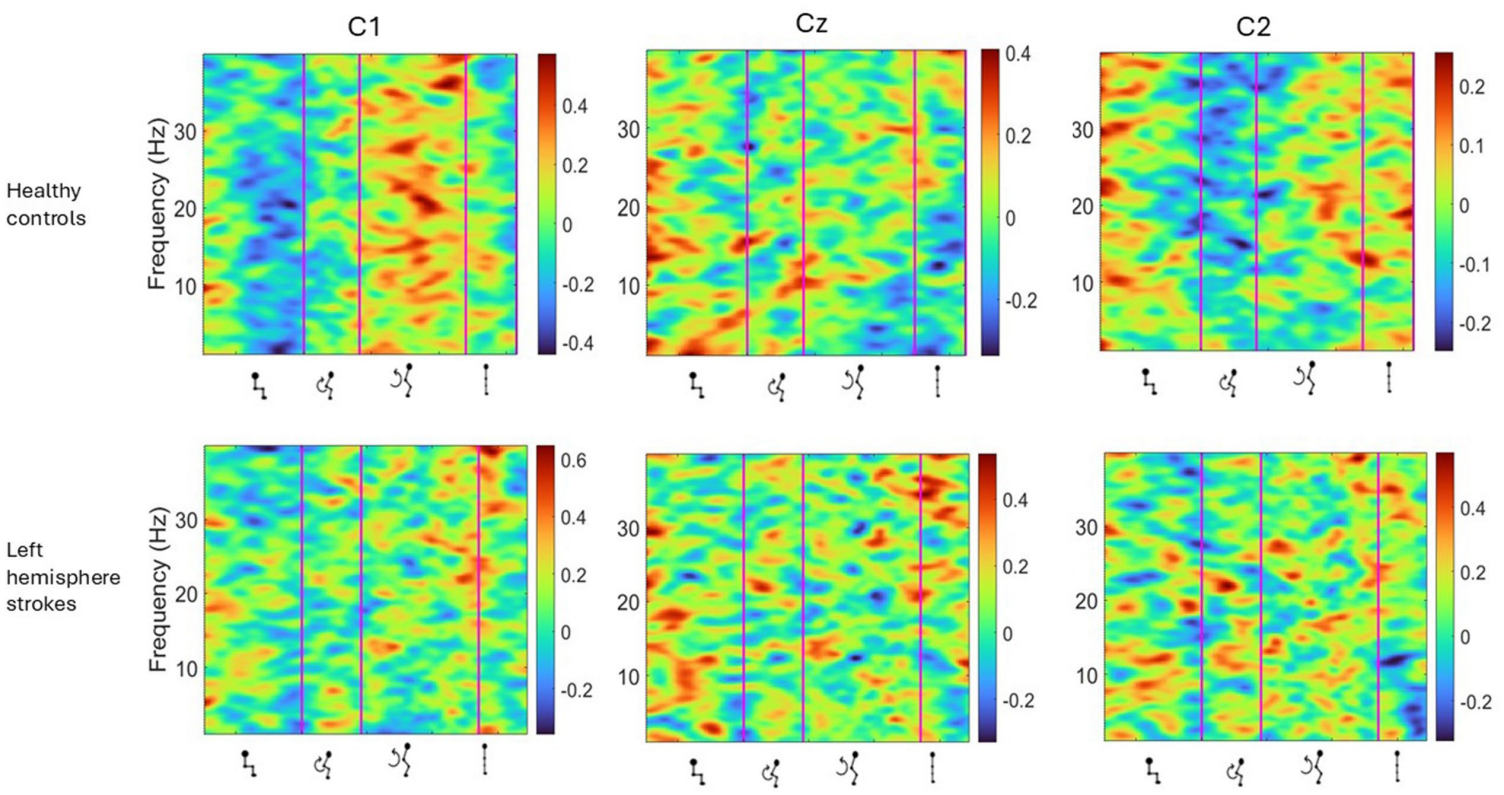
Average ERSP output for the Cz, C1 and C2 channels across all trials for all participants with left hemisphere strokes and separately for their matched healthy controls. Y-axis is frequency between 0-40 Hz, and in x-axis the time in ms and kinematic phases of standing up. Red and yellow spots represent synchronization, and darker blue spots represent desynchronization.

A statistically significant difference (*p* < 0.05) in ERSP was not found at group level (seven left hemisphere strokes verses seven matched healthy controls, Table 2) however some important differences were observed in grand average ERSP plots and between individual participants. As presented in Figure 3 using single participant ERSP, healthy controls exhibited consistent and well-defined desynchronization (ERD) in the beta (15–30 Hz) and alpha (8–12 Hz) bands during movement preparation and execution, followed by post-movement synchronization (ERS). Stroke Survivors however demonstrated inconsistent synchronization-desynchronization patterns across participants. Beta and alpha band ERD were delayed (by 0–2.2 s), reduced in magnitude (~0.2 dB) or absent in this group, particularly in the affected hemisphere (Figure 3). Healthy controls displayed bilateral but slightly dominant ERD/ERS in one hemisphere. In stroke survivors, asymmetric activity was observed with weaker ERD in the affected hemisphere. Healthy controls further exhibited a rapid return to baseline or increased beta ERS in the extension phase whereas stroke survivors showed prolonged and/or reduced ERS, in the affected hemisphere (Figure 3). Results were consistent in both right hemisphere and left hemisphere stroke. A similar pattern of synchronization/ de-synchronization is seen at population level for both groups in Figure 4 where a grand average of ERSP spectrogram across all seven left hemisphere strokes verses seven matched healthy control participants is presented.

**Table 2:** Comparison of the average ERSP between subjects with left hemisphere strokes and matched healthy controls, across alpha and beta frequency bands, for selected left hemisphere and central EEG channels.

EEG channel	Sitting	Flexion phase	Extension phase	Standing
Alpha				
Cz	W = 0.57 *p* = 0.58	W = 0.85 *p* = 0.42	W = 1.96 *p* = 0.08	W = 1.09 *p* = 0.30
C1	W = 1.59 *p* = 0.15	W = -0.14 *p* = 0.89	W = 1.52 *p* = 0.16	W = 2.11 *p* = 0.02
C3	W = 0.45 *p* = 0.66	W = 0.66 *p* = 0.52	W = 1.12 *p* = 0.29	W = 0.08 *p* = 0.29
Beta				
Cz	W = 1.54 *p* = 0.15	W = 1.17 *p* = 0.27	W = 1.23 *p* = 0.25	W = 0.71 *p* = 0.50
C1	W = 1.23 *p* = 0.25	W = 0.58 *p* = 0.58	W = 0.63 *p* = 0.55	W = 0.87 *p* = 0.39
C3	W = 0.62 *p* = 0.55	W = 0.17 *p* = 0.86	W = -0.44 *p* = 0.67	W = 0.70 *p* = 0.67

Highlighted boxes = *p* < 0.05.

‘W’ denotes to the Wilcoxon rank-sum test statistic and ‘*p*’ represents to the probability value.

Table 2 depicts the group level comparison of seven left hemisphere strokes verses seven matched healthy controls, across alpha and beta frequency bands, for selected left hemisphere EEG channels. A significant difference was observed between the left midline C1 electrode during the standing phase. A grand average for right-hemisphere and bilateral-hemisphere stroke participants was not computed, as the small sample size precluded reliable group-level analysis.

**Figure 5:**
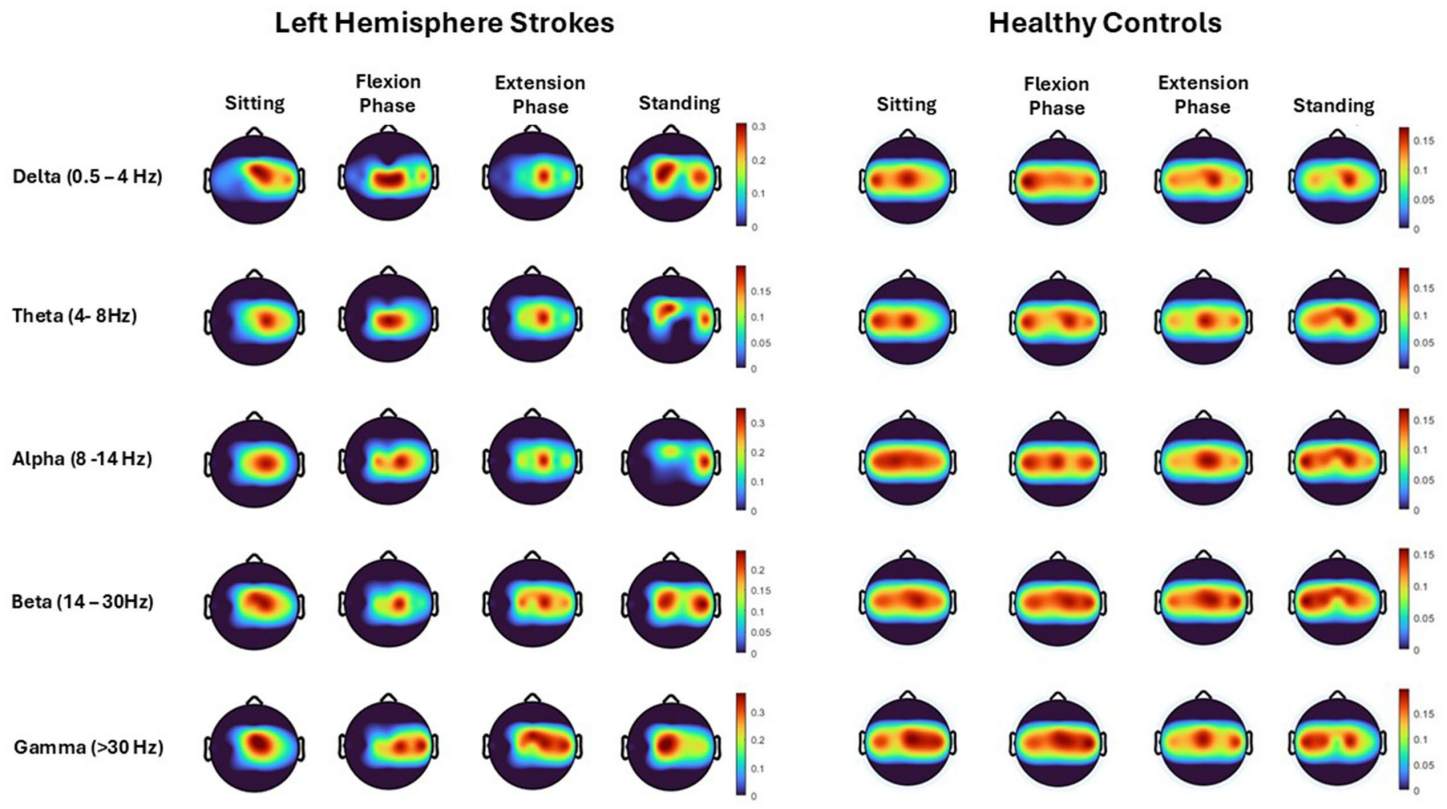
Topoplots show cortical activity (power spectral density), across each movement phase (sitting prior to movement, flexion phase, extension phase and upright standing) and each frequency band (Delta, Theta, Alpha, Beta, Gamma) averaged across all trials from all left hemisphere strokes (7 participants) and their matched healthy control participants.

Topographical mapping of the ERSP spectrogram identified the cortical areas that are most active during the phases of sit-to-stand across the population groups. Results include data in pre-movement sitting and in standing on termination of the sit-to-stand transfer and, as typical for EEG, is described in terms of rhythmic activity divided into the five frequency bands, delta (0.5- 4 Hz), theta (4–8 Hz), alpha (8–14 Hz), beta (14–30 Hz) and gamma (>30 Hz). A grand average of all left hemisphere strokes (7 participants) are presented alongside their matched healthy control participants (Figure 5). Results show that healthy control participants display bilateral but slightly dominant left hemisphere activity during all phases of sit-to-stand. Stroke survivors however show asymmetric cortical activation with substantially increased activation in the right hemisphere (the contralesional hemisphere). This is pronounced during sitting pre-movement and the flexion phase, although statistical comparison of inter-hemisphere ERSP (affected hemisphere compered to unaffected hemisphere) of the stroke group revealed no statistically significant differences (*p* < 0.05). Although not statistically significant larger interhemispheric discrepancies were evident in the Topoplots of stroke survivors with a higher level of impairment as indicated by the mRS.

### 3.4 Cortico-muscular coherence

**Figure 6:**
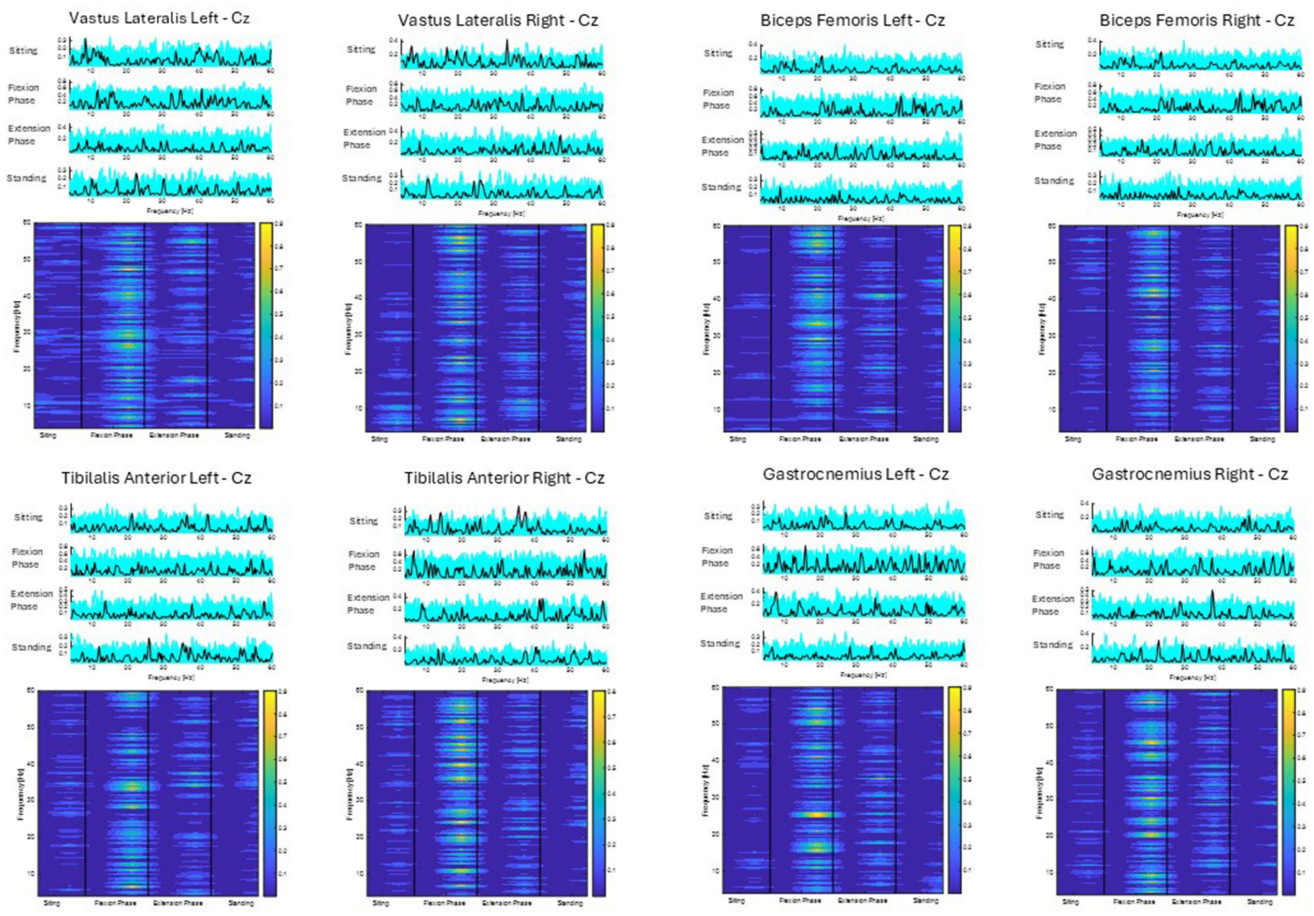
Corticomuscular coherence between the EEG recording at the Cz electrode and EMG recording of the vastus lateralis, biceps femoris, tibialis anterior and gastrocnemius muscles, respectively, across the phases of sit-to-stand. Data were in all cases pooled data from all repetitions from one post-stroke participant (P10) (15 trials). The upper plots show CMC across the frequency domain (black line), alongside 75 randomly generated surrogate coherence spectra (turquoise line). The lower plots show the color coded magnitude of coherence as indicated using color bars to the right of the plots.

CMC between the central Cz electrode and individual lower limb muscles (Vastus Lateralis, Biceps Femoris, Tibialis Anterior, Gastrocnemius Lateralis) are depicted for all 10 participants averaged across all sit-to-stand trials (Figure 6). Color plots depict higher coherence in the latter half of the flexion phase and in the extension phase across all muscle groups and all participants consistent with ERSP and EMG findings. However, as detailed in the methods section and presented visually in Figure 6, when CMC is plotted for each phase of sit-to-stand across frequency bands, alongside 75 randomly generated surrogate coherence spectra, the findings do not consistently exceed the chance of identifying corticomuscular coherence from randomly generated data. CMC (*p* < 0.05) was observed during the extension phase between the Cz electrode and vastus lateralis in three participants, biceps femoris in two participants and gastrocnemius in two participants. For the participant presented below (P10) statistically significant coherence (*p* < 0.05) was observed in the extension phase, between the Cz electrode and the bilateral gastrocnemius.

## 4 Discussion

This study, to the authors knowledge, is the first to co-register and report 3D kinematic, EMG and EEG data capture during sit-to-stand transfers in stroke survivors or any other neurological population. Consistent with previously published literature (Hsu et al., 2019; Hu et al., 2013), kinematic data confirm post-stroke participants take significantly longer to complete the sit-to-stand transfer compared to healthy controls (*p* = 0.038); however, they maintain the same kinematic movement phases and relative timing of phases (Hsu et al., 2019). Similarly, EMG data support the wider literature that confirms stroke survivors stand up using the same temporal muscle activation patterns as healthy controls, however peak activity of the vastus lateralis and biceps femoris are delayed, particularly for the hemiplegic side (Hsu et al., 2019; Hu et al., 2013; Hsu et al., 2023). The EEG data in this current study however, add new knowledge to our understanding of the higher-level control of sit-to-stand in a neurological population highlighting differences in cortical activity during the sit to stand transfer between healthy controls and stroke survivors particularly ERD/ERS patterns in the alpha and beta frequency bands and in the asymmetry between brain hemispheres.

### 4.1 Kinematics and muscle activity patterns

As noted, kinematic and EMG data are consistent with those previously reported in the existing literature (Hsu et al., 2019; Hu et al., 2013; Hsu et al., 2023). Firstly, the overall movement time of the individuals with hemiparesis is longer (Hsu et al., 2019; Hu et al., 2013), noted in this study to be almost 10% longer than healthy controls (Figure 1). Secondly, while there are no significant differences in the temporal parameters of muscle activity between participants with stroke and healthy individuals, as observed in Figure 2, delayed peak activation of lower-limb muscles is evident in individuals with hemiparesis, particularly in the unaffected side (Hsu et al., 2019; Hsu et al., 2023), meaning post-stroke patients favor their unaffected limb (Hsu et al., 2019; Hsu et al., 2023), This explanation is supported by literature that observes larger medio-lateral center of pressure displacement and altered weight distribution in the lower-limbs post stroke (Cheng et al., 1998; Boukadida et al., 2015) In this current study (Figure 2), a delay in contraction of Biceps Femoris and to a lesser extent Vastus Lateralis is observed post-stroke when compared to healthy controls, suggesting impairment of important anticipatory postural adjustment that is central in origin (Hsu et al., 2019). EEG data can for the first time support this hypothesis showing delayed alpha and beta ERD, particularly in the ipsilesional hemisphere. This finding is novel as it has not been previously reported in the literature.

EMG findings, confirming what is already known regarding muscle activation patterns, are clinically meaningful as asymmetrical muscle activity during sit-to-stand post-stroke is associated with a higher risk of falls (Figure 2) (Cheng et al., 1998). Moreover, individuals who demonstrate substantial motor recovery following stroke exhibit increased peak muscle activation across all synergies, with the most pronounced changes observed in the first synergy, corresponding to the momentum transfer phase (Wang et al., 2022). Notably, the timing of peak activation shifts earlier in the movement sequence (Wang et al., 2022; Yang et al., 2019). Correct timing of peak activity in the quadriceps and hamstrings muscle groups during the flexion phase of sit-to-stand could be considered as a potential rehabilitation target post-stroke based on the findings reported here and in the wider literature (Wang et al., 2022; Yang et al., 2019).

### 4.2 Power modulation differences

ERSP analysis in the current study reveals differences in cortical activity during the execution of a sit-to-stand transfer between healthy controls and stroke survivors, particularly in the power modulation of alpha and beta frequency bands (Figures 3, 4). Healthy Controls for example were observed to have well-defined ERD in the alpha (8–12 Hz) and beta (15–30 Hz) bands during movement preparation and early execution, followed by post-movement ERS. This power modulation pattern is typical of voluntary movements (Toro et al., 1994; Fry et al., 2016), recognized as reflecting efficient motor control and sensorimotor processing (Delcamp et al., 2024). It has previously been observed in other whole body movements such as gait (Borhanazad et al., 2024). This research group recently reported the same finding during the complex task of sit-to-stand for the first time in healthy individuals. These advances now provide an important baseline from which abnormal cortical activity that may be driving the known kinematic and electromyographical changes post stroke can be critically examined (McDonald et al., 2025). ERSP modulation during sit-to-stand execution in stroke survivors was not consistent across participants. Data evidenced that stroke survivors exhibited weaker ERD during the movement, particularly in the affected hemisphere, indicating impaired cortical activation (Delcamp et al., 2024; Tangwiriyasakul et al., 2014). Comparison of these findings to the wider literature in stroke is limited as the majority of post-stroke ERSP studies focus on the upper limb (Delcamp et al., 2024; Tangwiriyasakul et al., 2014). However, a limited number of studies analyzing post-stroke gait have shown weaker alpha ERD compared to healthy controls (Mayor-Torres et al., 2022; Ko et al., 2021) and furthermore that increased impairment correlated with decreased alpha ERD during the stance phase of gait (Ko et al., 2021).

### 4.3 Asymmetry between hemispheres

This study identified asymmetrical cortical activation in stroke survivors during pre-movement and sit-to-stand phases, not previously reported in the literature (Figures 3–5). Despite the stroke survivors included in this study having low levels of disability (mRS < 3) and functional impairment, nonetheless they demonstrated increased cortical activation in their unaffected hemisphere compared to their affected hemisphere, as displayed by increased power spectral density (Figure 5) and asymmetrical ERSP plots (Figure 3). While the degree of asymmetry varied across individuals, when considered on a case-by-case bases, individuals with higher levels of impairment (mRS 2/3) appear to have the largest asymmetries. In addition, all but one stroke participant were in the chronic stage of stroke recovery (>6 months post-stroke), while one participant was in the late-subacute phase (>12 weeks post-stroke) (Li et al., 2024). It has been shown in comparator gait studies that ERD and ERS modulation vary with the level of gait impairment and with stroke chronicity, with less impairment and more chronic stroke show more typical modulation patterns (Ko et al., 2021; Oliveira et al., 2018; Carino-Escobar et al., 2019). Furthermore, upper limb research has shown that higher Brain Symmetry Index values, reflecting more power asymmetry over the hemispheres, predict more motor impairment 6 months after stroke (Saes et al., 2021). As the first study to describe the presence of hemispheric asymmetries during sit-to-stand, these findings now warrant further examination. This study included participants only in the chronic phase of stroke, many of whom had low level of impairments and some of whom had bilateral stroke. Examination in the acute and subacute phases of stroke is now warranted alongside stratification of participants by impairment levels.

### 4.4 Corticomuscular coherence during sit-to-stand post stroke

This research group recently reported CMC during sit-to-stand in healthy controls was inconsistent across participants and statistically significant coherence was observed in only a subset of healthy participants (McDonald et al., 2025). The lack of consistency in findings was attributed to several challenges associated with measuring CMC during dynamic whole-body movements. CMC has been shown to be lower in posturally focused, position-control tasks than in force-control tasks (Poortvliet et al., 2015). Studies examining CMC during isokinetic muscle contractions, such as those occurring in sit-to-stand transitions, are limited. The majority of published research focuses on isometric contractions which typically produce higher levels of coherence (Liu et al., 2019). Therefore, it was not surprising that inconsistent CMC patterns were observed in post-stroke participants. A notable finding warranting further investigation was that, in the subset of post-stroke participants exhibiting significant coherence, this coherence was predominantly observed in the quadriceps and hamstring muscle groups during the extension phase of the sit-to-stand movement. This contrasts with prior observations in healthy control participants, wherein significant CMC was more frequently detected during the flexion phase of the movement in the same muscle groups (McDonald et al., 2025). This may represent the delayed peak EMG activity of the quadriceps and hamstrings muscles during the flexion phase of sit-to-stand observed in the stroke participants. As a result of challenges assessing CMC during this complex movement no firm conclusions can be drawn from these findings given the intersubject variations in both healthy and stroke study participants.

### 4.5 Implications for rehabilitation and robotics

Reduced ERD in stroke survivors evident in this study during sit-to-stand suggests impaired motor cortex activity which could prove a useful biomarker for recovery and/or a target for rehabilitation strategies such as neurofeedback or brain-computer interface technologies (BCIs). Neural (EEG) interfacing technology could offer new strategies for lower-limb robotic rehabilitation by promoting more active engagement in movement intent and/or neurophysiological feedback. In the upper limb, Human Machine Interface robotic therapy is proven to enhance motor outcomes in neurological populations such as stroke (Cervera et al., 2018). Furthermore, extensive literature detail approaches for decoding upper-limb kinematics or muscle activity using cortical recordings toward brain machine interface applications (Camargo-Vargas et al., 2021). Similar approaches to the lower-limb remain scarce where disappointingly, the same technology has failed to realize similar outcomes for lower-limb motor recovery. No intent-controlled Robotic Assisted Gait Training has been identified in clinical studies (Sarasola-Sanz et al., 2017) or in the stroke rehabilitation sector (Lennon et al., 2020). Limited research examining robotic sit-to-stand assistance exists and has, in the main, focussed on human intention (Li et al., 2021; Romano et al., 2017). This study offers important new information for the development of responsive BCI sit-to-stand rehabilitation devices for this population, highlighting potential difficulties in variable activity both between participants and within participants as they make functional improvements and suggests the need to train BCI systems at an individual level.

### 4.6 Limitations

Results of this study must be interpreted with a degree of caution. The relatively small size of the stroke group and healthy counterparts limits statistical power and the ability to draw strong conclusions. Correction for multiple comparisons was not applied as the analysis was limited to 10 focused comparisons, aimed at guiding future hypothesis-driven research. However, this approach increases the risk of Type I errors due to the relatively large number of statistical tests conducted. Therefore, the results should be interpreted with appropriate caution. In effect, heterogeneity of findings across individuals and kinematic stages of sit-to-stand indicate a fundamental challenge for development of future brain-computer interface devices that can accurately address sit-to-stand movements.

The stroke group exhibited a significantly higher BMI compared to the control group (*p* = 0.037). Elevated BMI can attenuate both EMG and EEG signals due to increased adipose tissue, which may affect electrode impedance on the scalp and over muscles (Farina et al., 2002). However, we do not believe this difference substantially impacted the current findings. Only 2 out of 10 stroke participants were classified as having severe obesity (defined as BMI ≥ 35.0) (World Health Organization, 2000) indicating a level of adiposity that could meaningfully affect signal quality. As such, a subgroup analysis was not performed.

Future research should include a larger sample of stroke participants encompassing a broader range of impairment levels and varying times post-stroke to enhance generalisability. Additionally, while increasing the number of repetitions is challenging in this population due to fatigue, distributing repetitions across multiple testing sessions over several days could improve statistical power to detect meaningful effects.

A limitation of the peak-within-task normalization approach employed is that, while it reduces inter-participant variability and facilitates within-muscle temporal analyses and characterization of activation patterns, it does not permit valid inter-limb comparisons. The ideal approach, and current best practice, is to normalize to a maximum voluntary contraction (MVC) matched to the task (Besomi et al., 2019); however, this is challenging to implement for sit-to-stand movements due to their dynamic nature and the substantial changes in muscle length that occur throughout the movement. This approach should be considered in future studies.

## 5 Conclusion

The collection and analysis of co-registered kinematic, EMG, EEG data during this study facilitates, for the first time, a systemic understanding of the neurophysiological bases of the complex sit-to-stand movement in a neurological population with stroke. The results provide novel insights into the neural top–down control of physiological function in this population.

The results highlight differences in cortical activity between healthy controls and stroke survivors particularly in ERD/ERS patterns in the alpha and beta frequency bands and in the asymmetry largely evident between brain hemispheres in stroke. Results suggest slower onset of ERD and lower power during the execution of sit to stand following stroke, even in individuals with low levels of disability and in the chronic phase of stroke. This study offers important information for the development of responsive sit-to-stand rehabilitation devices for this population, confirming for the first time that cortical activation patterns during sit-to-stand are altered in stroke survivors. This provides new perspectives and challenges for the development of BCI sit-to-stand rehabilitation devices for this population, highlighting variable activity both between participants and potentially within participants as their function improves.

## Data Availability

The raw data supporting the conclusions of this article will be made available by the authors, without undue reservation.
