# Supplementary material for: Neurophysiological insights into sit-to-stand post stroke
Source: Front Neurosci. 2025 Sep 2;19:1646498. doi: 10.3389/fnins.2025.1646498 (PMC12436286; doi:10.3389/fnins.2025.1646498)
Supplement: Supplementary file 1 [file Data_Sheet_1.docx]

Table S1: Dataset details

| Participant | Number of sit-to-stand transitions | Channels excluded due to artefact * |
| --- | --- | --- |
| Stroke survivors | | |
| 1 | 16 | C6 |
| 2 | 15 | C2, C4 |
| 3 | 18 | - |
| 4 | 6 | C1, C3, C4, C5, C6 |
| 5 | 9 | - |
| ~~6~~ | ~~0~~ | - |
| 7 | 6 | - |
| 8 | 15 | - |
| 9 | 15 | - |
| 10 | 15 | C3, C5 |
| 11 | 18 | - |
| Healthy Controls | | |
| 1 | 75 | - |
| 2 | 30 | Cz for 15 trials |
| 3 | 65 | - |
| 4 | 40 | Cz, C4, C6 |
| 5 | 70 | C1, FCz |
| ~~6~~ | ~~70~~ | - |
| 7 | 30 | - |
| 8 | 20 | C2 |
| 9 | 70 | - |
| 10 | 75 | - |
| 11 | 45 | - |

Table S2: Characteristics of participants with a diagnosis of stroke

| Participant | Sex  (Male/ Female) | Age | Height | Weight | Stroke type  (Ischemic/ Haemorrhagic) | Stroke laterality  (Right/ Left) | Time since stroke (years) | mRS | FAC | COGTEL Score | Mobility aids |
| --- | --- | --- | --- | --- | --- | --- | --- | --- | --- | --- | --- |
| 1 | F | 50 | 163.5 | 130 | Ischemic | Right | 9 | 1 | 5 | 15 | - |
| 2 | F | 36 | 180 | 108 | Ischemic | Left | 0.3 | 1 | 5 | 25 | - |
| 3 | F | 38 | 180 | 82 | Ischemic | Left | 6 | 1 | 5 | 43 | - |
| 4 | F | 42 | 172 | 98 | Haemorrhagic | Right | 6 | 2 | 4 | 8 | - |
| 5 | F | 33 | 165 | 102 | Ischemic | Left | 2 | 2 | 5 | 15 | - |
| ~~6~~ | ~~M~~ | ~~54~~ | ~~177~~ | ~~73~~ | ~~Haemorrhagic~~ | ~~Bilateral~~ | ~~7~~ | ~~4~~ | ~~1~~ | ~~13~~ | ~~Frame~~ |
| 7 | M | 34 | 179 | 99 | Ischemic | Left | 2 | 1 | 5 | 33 | - |
| 8 | M | 55 | 180 | 108 | Haemorrhagic | Left | 3 | 3 | 4 | 7 | AFO Right |
| 9 | F | 40 | 162.5 | 72 | Ischemic | Left | 8 | 1 | 5 | 27 | - |
| 10 | F | 60 | 170 | 65 | Ischemic | Left | 8 | 2 | 5 | 23 | - |
| 11 | M | 55 | 190 | 119 | Haemorrhagic | Bilateral | 3 | 1 | 5 | 22 | - |
|  | 4 Male/ 8 Female | 44.4 ± 9.3 |  |  |  |  | 4.8 ± 2.8 |  |  |  |  |

mRS= modified Rakin Scale, FAC= Functional Ambulation category, COGTEL= Cognitive Telephone Screening Instrument, AFO= Ankle Foot Orthosis.
